# Correlations of Symmetry and Frequency in Alphabets and in Natural Systems

**DOI:** 10.3390/e28101066

**Published:** 2026-09-28

**Authors:** Edward Bormashenko, Shraga Shoval, Mark Frenkel, Michael Nosonovsky

**Affiliations:** 1 Department of Chemical Engineering, Ariel University, P.O. Box 3, Ariel 407000, Israel; edward@ariel.ac.il (E.B.); markfr@ariel.ac.il (M.F.); 2 Department of Industrial Engineering and Management, Faculty of Engineering, Ariel University, P.O. Box 3, Ariel 407000, Israel; shraga@ariel.ac.il; 3 Department of Mechanical Engineering, University of Wisconsin-Milwaukee, Milwaukee, WI 53211, USA; 4 Department of Biology and General Genetics, Sechenov First Moscow State Medical University, Izmaylovskiy Boulevard 8, Moscow 105043, Russia

**Keywords:** symmetry, graphemes, Shannon entropy, letter frequency, numerical figures, Spearman correlation, Kendall correlation, Benford’s law, colloidal clusters, type–token relationship

## Abstract

We investigate the relationship between the graphical symmetry of symbols and their frequency of occurrence in culturally evolved symbolic systems and compare the observed tendencies with those found in physical structures. Capital letters of the English and Russian alphabets were classified according to their invariance under vertical reflection, horizontal reflection, and rotation through 180°. The graphical symmetry of numerical figures was analyzed across four numeral systems. Symmetry was examined for 1000 Japanese Kanji arranged into frequency-ranked groups. Spearman’s and Kendall’s rank-correlation tests revealed positive symmetry–frequency tendencies in both alphabets, although the associations did not reach conventional statistical significance. A strong and statistically significant positive association was obtained for the average symmetry of the numerical figures and their leading-digit frequencies predicted by Benford’s law. The Japanese Kanji produced the strongest statistical result. Despite their different cultural origins and graphical organization, the investigated alphabets and numeral systems exhibited surprisingly similar symmetry-class entropies, suggesting a possible common balance between graphical regularity and symbol distinguishability. For comparison, seven-bond and eight-bond colloidal clusters demonstrated the opposite tendency, with asymmetric configurations occurring more frequently and dominating their symmetry-class distributions.

## 1. Introduction

The concept of symmetry is located at the intersection of cognitive science, mathematics and physics [1,2,3]. In the classical experimental research by Bornstein et al., it was demonstrated that babies tended to view mirror/symmetrical images, especially lateral mirror images, as equivalent stimuli [1]. Symmetry provides perceptual constancy of images to be treated by the human brain [2]. Perceptual constancy refers to the fact that the perceived geometrical parameters of objects remain constant despite transformations of the items such as their displacement/rotation or deformation. Perceptual constancy is important in a vast majority of kinds of human activities [2,4], like recognition of items, patterns and images, terrain orientation, reading of texts, etc. Perceptual constancy would not exist without the spatial and temporal stability of objects, including their dimensions and shape [2,4]. Formally, spatial and temporal stability of objects is addressed in mathematics and physics as invariants, also known as symmetries [5,6,7]. In attempting to judge whether a briefly presented rotated figure was symmetrical (circle or square) or asymmetrical (ellipse or rectangle), a bias towards perceiving symmetry was revealed [8]. Thus, it is reasonable to suggest that there is also a bias towards symmetric symbols representing graphemes in various alphabets. Symmetry makes recognition of letters easier [9]. We hypothesized that frequency of symmetrical letters in texts is higher than that of non-symmetrical ones; in other words, the frequency of the symbol/grapheme is expected to be dependent on its symmetry. We studied this dependence for various sets of graphemes, and we will compare this dependence with the abundance of symmetrical structure in nature.

We will consider a correlation between the symmetry of symbols used in various symbolic systems, both natural and artificial, and their frequency. A symbol is a visual image or sign representing an idea, object, or relationship. A symbolic system is an organized collection of symbols and rules for their use that can carry certain meanings. Examples of artificial symbolic systems are alphabets and numerals, while natural symbolic systems, such as the DNA code, are often found in biological systems.

Alphabets used for computer fonts are sets of letters/graphemes that represent sounds or phonemes of a spoken language [10,11]. From the semiotic point of view, the type-token distinction of the alphabetic symbols is often made. The type represents a particular letter as a symbol in general, while a token is a particular instance of the letter [10,11]. For example, the word “letter” uses only four types of letters: “l”, “e”, “t”, and “r”. At the same time, it uses six tokens of letters, since the letters “e” and “t” are repeated twice. Like every set of symbols used for information encoding, an alphabet can possess a certain hidden structure, because elements of alphabets, i.e., letters, have certain relations between themselves. One kind of such relation is statistical relations such as the frequency distribution of symbols. It has been shown by various experimental studies that the frequency distribution of letters in most texts written in a natural language tends to follow the power or Zipf distribution law [12,13,14]. Such an observation applies to mentions of letters, i.e., to letters as tokens.

Another characteristic of symbols is their graphical complexity. While it is difficult to quantify such an elusive quality as complexity, recently, attention to the symmetry of alphabetic symbols has been paid [9,15]. There are various suggestions of what can be behind the symmetry of letters including the mythological concepts behind the horizontal mirror symmetry (e.g., reflecting the symmetry between the earthy and underground worlds) and the role of symmetry in the evolution of the writing system [16,17,18]. The graphical symmetry considerations of symbols apply to letters as types.

Numerals also represent symbolic systems, such as the set of digits 0, 1, … 9. Surprisingly, for many types of data, the statistical distribution of digits is uneven and it is governed by Benford’s law [19,20,21]. According to this law, which applies to digits from 1 to 9, the frequency of finding a digit decreases logarithmically with increasing its numeric value. Thus, the chance of finding the digit “1” in any random set of data is about 30.1%, while the chance of finding the digit “9” is only about 4.6%.

Symmetry of symbols/graphemes and grammatical structures of natural languages is becoming an important topic of investigation in information processing studies and cognitive science [9]. Tanchip et al. suggested a methodological framework for inferring symmetry of verb predicates in natural language using a feature-based and a context-based approach [22]. The category of symmetry has also emerged in the sign language [23]. Symmetric predicates are related to the semantics of reciprocal alternations (most symmetric predicates in English show a simple kind of reciprocity) [24]. In this context and given the trend to relate the study of symmetry and similarity to cognition [9,25], an investigation of symmetry in alphabetic and numerical symbols is of particular interest. For the number of inscriptions, vertical mirror symmetry of the signs is recognized [26]. Many ancient scripts were written in a boustrophedon style, i.e., with alternating directions [26]. This means that the writer wrote the first line from left-to-right, then reversed the direction to right-to-left in the second line, and then kept switching the direction for each successive line of the inscription. As a consequence of that, the boustrophedon inscriptions often used a vertical mirror-symmetric version of the usual sign recognized [26].

It was demonstrated recently that, compared to phonemes, letter shapes require more binary features for a complete description [27]. These features are also less informative in letters than in phonemes: the chances that two random letters in a script differ on any given feature are low. Letter shapes, which have more degrees of freedom than speech sounds, use those degrees of freedom less efficiently.

In this context, it is important to emphasize the importance of the “oblique effect” in writing systems; it was reported that vertical and horizontal orientations are preferably treated by human vision, and vertical symmetry is favored over horizontal symmetry [9,28,29,30]. Thus, the symmetry in writing systems turns out to be not only a physical/mathematical phenomenon but also a human factor [15].

We hypothesize that there is a correlation between the statistical frequency of a letter as a token and its symmetry as a type. This is because more frequently used symbols would tend to be either simple or more symmetric. In the following sections, we will study symmetry-frequency correlations of two alphabets (Russian and Latin) and of several graphical systems of numerals, and we will compare the results with similar correlations in artificial systems, such as small colloidal clusters and ligands.

## 2. Materials and Methods

### 2.1. Latin and Russian Alphabets

The capital letters of the Latin alphabet and Cyrillic Russian alphabets were analyzed from the viewpoint of the presence of symmetry groups, including the horizontal and vertical mirror symmetry and the central symmetry. The number of symmetry operations for English (Latin) and Russian (Cyrillic) capital letters are shown in Table 1. Note that the classification of graphical symmetry is dependent on the particular graphical representation of a symbol. The computer font Palatino Linotype (in which this paper is written) was analyzed for the Latin capital letters; the computer font Franklin Gothic Book was analyzed for the Cyrillic capital letters.

The frequency data of Latin letters in modern English were obtained from the data available in the literature and summarized in Table 2 for the 26 letters of Latin alphabet [31]. Note that the frequency in texts, frequency in dictionaries, and frequency of the first letter can differ. We relied on the frequency in texts as the most relevant for our study.

The frequency data were obtained from the Russian National Corpus (RNC) and from Wikipedia (https://en.wikipedia.org/wiki/Letter_frequency, accessed on 23 April 2017) [32] (in Russian). These frequencies are presented in Table 3 for 32 letters of modern Russian alphabet (excluding the letter Ё, which is not used consistently in modern spelling).

### 2.2. Numerals

We studied four types of numerals belonging to different cultures, namely: (i) modern western numerical digits (often referred to as “Hindu–Arabic numerals”) from “1” to “9” (digit “0” was excluded since it is usually not included into Benford’s law), (ii) Roman numerals from “I” to “IX,”, (iii) Eastern Arabic numerals from ١ to ٩, and (iv) Chinese numerals, which are also used by many Asian cultures, such as Japanese. Note that for symmetry considerations, certain simplifications were made. Thus, for the digit “1” the form as a vertical line “I” was used, so that the digit is invariant relative to two symmetry operations: the mirror symmetry against the vertical and horizontal lines. The Arabic sign “١” was treated in the same way. The frequency data were obtained from Benford’s law which states that the leading digit *d* occurs with the probability

(1) $$P(d)=\log_{10}\left(1+\frac{1}{d}\right).$$

This data is summarized in Table 4.

### 2.3. Japanese Kanji Characters

For comparison, we also studied the list of 1000 most frequent Japanese Kanji characters based on the frequency list of [33]. The methodology for analyzing the Kanji script was different from that of the Latin and Cyrillic scripts. The reason for that is that the Japanese system of writing is very different from the Western one, and, in particular, the number of symbols in Japanese script is significantly greater than in Western scripts. Mirror symmetry is the most common type for Kanji signs. It prevails over other types of symmetry. The list was divided into 25 groups of 40 characters each, and symmetry was counted. The results are presented in Table 5.

## 3. Results

### 3.1. Correlation Between Frequency of Letters and Their Symmetry in Russian and English

The correlation of the number of symmetry operations ranging from *N_SYM_* = 0 to *N_SYM_* = 3 (Table 1) and frequencies of letters, *P*, in English (Table 2) and Russian (Table 3) languages was determined.

As one can observe from Figure 1, in both cases more symmetric letters tend to be more frequent.

The linear trend line equation is given by

(2) $$P\left(N_{SYM}\right)=1.57N_{SYM}+2.26\;(\mathrm{English}),$$

(3) $$P\left(N_{SYM}\right)=2.95N_{SYM}+0.50\;(\mathrm{Russian}).$$

Note that while the slope of the trend-line is higher for Russian (2.95 as opposed to 1.57 for English), the coefficient of determination has an opposite trend, $R^{2}=0.899$ (English) and $R^{2}=0.628$ (Russian).

In the case of the Russian alphabet, two relatively infrequent but symmetric letters, “Х” and “Ж”, constitute outlier points. If these two points are removed, the coefficient of correlation becomes significantly higher, $R^{2}=0.976$. However, both letters are present already in earliest versions of Cyrillic alphabets in their symmetric forms with “Х” originating from the Greek letter χ and “Ж” being created for the Cyrillic alphabet to represent forms of the verb “жити” (“to live”). Therefore, there is no ground for removing these two letters from the consideration.

A possible explanation of the observed trend is that frequently used letters tended to be simplified, which resulted in more symmetric forms. Symmetry of graphemes provides parsimony of graphical information necessary for the drawing of the entire set of graphemes constituting the alphabet [17,34]. And it also provides the legibility of the texts [9]. These considerations at least partially explain the trend, illustrated with Figure 1.

### 3.2. Correlation Between Frequency of Numerals and Their Symmetry

In a similar way, the numerals were studied. For the four types of numerals (Table 4), average symmetry was calculated and correlated with Benford’s Law frequency (Equation (1)) as summarized in Table 6.

The correlation was found (Figure 2) with the trend line equation given by

(4) $$P\left(µ\right)=11.34µ+2.60$$

and the coefficient of determination $R^{2}=0.740$. We can conclude that, similarly to alphabets, more frequent numerals tend to be more symmetric.

Again, symmetry of the symbols representing the numerals provides parsimony of graphical information necessary for their drawing and the legibility of the numerical data.

### 3.3. Kanji Characters

For *Kanji* characters, the vertical mirror symmetry was investigated. We plotted the relative frequency of symmetric characters (%) vs. the group number, *n*, where each group included 40 characters. The total of 1000 most frequent characters were divided into 25 groups. The correlation was found (Figure 3) with the trend line equation given by

*f*(*n*) = −0.7692*n* + 21.9 (5)

Clearly, the trend was that less frequent characters are unlikely to be symmetric, although the value of the coefficient of determination was only $R^{2}=0.57$. The value of $R^{2}$ is low; thus, Spearman and Kendall statistical analysis should be applied for the data related to Japanese Kanji characters. The results of Spearman and Kendall tests are reported below in Section 3.5.

### 3.4. Comparison of Artificial Symbolic Systems and Natural Systems

It is interesting to compare artificial symbolic systems—alphabets and numerals—with natural systems such as small colloidal clusters and ligands [35,36,37,38,39]. An example of such system is acoustically levitating small clusters of spherical colloidal particles. For a system of six particles, Perry et al. [39] identified a number of seven-bond and eight-bond configurations for 2D clusters. Frequency distributions of these configurations tend to follow the Zipf law (Figure 4). Symmetry of such configurations can be quantified in a similar way to alphabetic and numerical symbols.

We calculated the dependency of frequency distribution vs. symmetry for the seven-bond and eight-bond colloidal clusters. The results are presented in Table 7. It is observed that the trend is opposite to the one found with alphabets and numerals. Symmetric structures are less frequent than asymmetric structures. Note that unlike cluster of rigid microparticles, whose interaction is dominated by hardcore repulsion, levitating micro-droplet clusters may possess new symmetries absent from rigid clusters. For example, the applicability of the 40-fold, 5-fold, and 7-fold symmetries and mathematical ADE-classification or the so-called simply laced Dynkin diagrams for small levitating droplet clusters have been suggested [35].

An explanation of this observation is that, while the cluster is in equilibrium, every possible way to build a cluster (having the same binding energy) occurs with the same probability. Consequently, there are fewer ways of building symmetric clusters. This is considered an entropic effect, since free energy decreases with increasing entropy according to ∆G=∆H−T∆S∆H=0, so that at constant temperature (*T* = const) and binding energy (∆H=0), less symmetric configurations are more favorable [40,41].

Another way of interpreting this effect is that symmetric systems have a smaller volume of configurational space and consequently they are less mobile. Shityakov et al. studied the ligand–receptor interactions of 1144 ligands, which are candidates for anti-COVID drugs [42]. They investigated the correlation between the Voronoi entropy, a measure of orderliness, of 2D ligand molecules and their affinity to receptors. They showed that less ordered ligands have higher mobility of molecular groups and therefore a higher probability of attaching to receptors (Figure 5).

From these results, we can observe that “symbol-like” natural structures, such as colloidal clusters and ligands tend to behave differently than artificial symbols used by human language. For natural systems, symmetric structures have fewer variants than corresponding non-symmetric structures (or lower entropy), consequently, they are less frequent. In artificial systems of symbols, the trend is opposite: more common symbols are more symmetric to minimize efforts needed to write them.

Every symbol combines two aspects: its form and its meaning. Iconicity is a similarity or analogy between symbols form and meaning [43]. It was argued that icons evolve into symbols as a consequence of the systematic shift in the locus of information from the sign to the users’ memory of the sign’s usage supported by an interactive grounding process [43,44]. Evolution of graphemes towards more symmetrical shapes provides the better efficiency of transfer of information from grapheme to the users’ memory [17].

In the Chomskian generative linguistics, it was believed that the iconicity is not a significant factor that affects functioning of human language, in particular, that the rules of syntax are independent of semantics (“Autonomy of Syntax”) [45]. Contrary to that, Cognitive Linguistics establishes that there is influence of semantics upon syntaxes of language [46]. Our study shows properties of a symbol as a type, such as symmetry of its form, and properties of a symbol as a token, such as frequency of use, correlate with each other.

### 3.5. Rank Correlation Analysis of Letters and Numerical Figures

Since the symmetry parameters $N_{SYM}$ and μ assume only a limited number of discrete values and consequently contain numerous ties, the relationships between symbol symmetry and frequency were additionally examined using two non-parametric methods: Spearman’s rank correlation coefficient $\rho_{s}$ and Kendall’s rank correlation coefficient $\tau_{b}$ (for more details see Appendix A). Every individual letter or numerical figure was treated as a separate observation.

Spearman’s coefficient evaluates the strength and direction of a monotonic relationship between two variables [47]. Kendall’s coefficient is based on comparisons between all possible pairs of observations [48]. A pair is concordant when the more symmetric symbol is also more frequent and discordant when the more symmetric symbol is less frequent. Kendall’s $\tau_{b}$ was used because this form of the coefficient corrects for tied values of the symmetry parameter. All reported values correspond to two-sided tests.

For the 26 letters of the English alphabet, Spearman’s analysis demonstrated a positive association between the symmetry score $N_{SYM}$ and letter frequency: $\rho_{s}=0.337,\;p=0.092.$ Kendall’s analysis produced a consistent result: $\tau_{b}=0.268,\;p=0.094.$ For the 32 letters of the Russian alphabet, excluding the letter Ё in accordance with the frequency dataset, Spearman’s coefficient was $\rho_{s}=0.281,\;p=0.120,$ whereas Kendall’s coefficient was $\tau_{b}=0.245,\;p=0.083.$ Thus, both rank correlation methods yielded positive coefficients for both alphabets. The direction of the associations is consistent with the proposed hypothesis that more symmetric letters tend to occur more frequently. However, none of the alphabetic associations reached the conventional statistical significance threshold of p<0.05 in the two-sided tests. The results for the English and Russian alphabets should therefore be interpreted as indicating a positive tendency rather than providing conclusive statistical confirmation of the symmetry–frequency relationship at the level of individual letters. The same analyses were performed for the nine digits shown in Figure 2. In this case, the average symmetry parameter μ, calculated from the four numeral systems presented in Table 4, was correlated with the corresponding first-digit frequency predicted by Benford’s law. Spearman’s rank correlation yielded $\rho_{s}=0.760,\;p=0.0176,$ whereas Kendall’s rank correlation yielded $\tau_{b}=0.667,\;p=0.0148.$ Both rank correlation tests therefore demonstrated a strong, positive, and statistically significant monotonic association between the average symmetry parameter of the numerical and their frequencies according to Benford’s law. Numerical figures possessing greater average graphical symmetry tend to correspond to more frequent leading digits. The results of the non-parametric analyses are summarized in Table 8.

These individual-symbol analyses complement the linear regressions based on the mean frequencies of the symmetry classes. The class-averaged alphabetic data yielded coefficients of determination of $R^{2}=0.899,$ for English and $R^{2}=0.693$ for Russian. The regression for the numerical figures yielded $R^{2}=0.740$. Note that these values are based on a small number of data points (*n* = 4).

The linear regressions indicate that the mean frequency increases with the symmetry parameter. However, Spearman’s and Kendall’s analyses do not assume a linear relationship and, in the case of the alphabets, retain the considerable variability among individual letters belonging to the same symmetry class. The rank correlation tests therefore provide a more conservative evaluation of the proposed symmetry–frequency relationship. Taken together, the regression and rank correlation analyses demonstrate a consistently positive symmetry–frequency trend in all three symbolic datasets. For the English and Russian alphabets, the rank correlations are positive but do not reach statistical significance when individual letters are analyzed using two-sided tests. For the digits, by contrast, the positive relationship is strong and statistically significant according to both Spearman’s and Kendall’s tests.

For the Japanese Kanji, the 1000 characters were divided into 25 consecutive frequency-ranked groups of 40 characters, as presented in Table 5. Each group was treated as one observation, and the number of vertically mirror-symmetric characters in the group was correlated with its frequency order. To maintain the same directional convention as that used for the alphabetic and numerical systems, the frequency-order variable was coded so that larger values corresponded to greater frequency. Spearman’s analysis demonstrated a strong positive association between Kanji frequency and vertical mirror symmetry, $\rho_{s}=0.770,\;p=6.72\times 10^{-6}.$ Kendall’s analysis, using $\tau_{b}$ to account for tied symmetry counts, yielded a consistent result, $\tau_{b}=0.635,\;p=1.74\times 10^{-5}$. Thus, both non-parametric tests demonstrate a strong and statistically significant concentration of vertically mirror-symmetric Kanji among the more frequently used characters. This result is based on frequency-group aggregates rather than on the individual frequencies of all 1000 Kanji and should be interpreted accordingly.

### 3.6. Shannon Entropy of the Symmetry Distribution

Shannon entropy was calculated as

(6) $$H_{class}=\sum_{k}p_{k}lnp_{k},$$

where $p_{k}$ is the fraction or probability belonging to symmetry class $N_{SYM}=k.$ The normalized Shannon entropy was calculated as

(7) $$H_{norm}=\frac{H_{class}}{lnK},$$

where *K* is the number of symmetry classes considered. An intriguing result of the Shannon analysis is the comparatively narrow range of symmetry-class entropies obtained for the English and Russian alphabets and for the Chinese, Western Hindu–Arabic, Roman, and Eastern Arabic numeral systems (Table 9). Despite their different cultural origins, historical development, and graphical organization, the corresponding values lie within the interval

(8) $$H_{class}≅0.85-1.10nats.$$

The normalized entropies also occupy a relatively restricted intermediate-to-high range, from 0.754 for the English alphabet to 0.878 for the Eastern Arabic numerals, while the Chinese numerals exhibit the maximally uniform value of unity. This convergence may reflect a common functional compromise operating in culturally evolved graphical systems. Symmetry promotes graphical economy, reproducibility, perceptual stability, and rapid recognition. Conversely, an excessive concentration of symbols in highly symmetric classes could diminish their graphical distinctiveness and increase the probability of confusion between different symbols. Alphabetic and numeral systems may therefore evolve toward repertoires containing both symmetric and asymmetric forms, producing moderately broad symmetry-class distributions. In addition, the geometry of planar glyphs restricts the readily realizable exact transformations mainly to vertical reflection, horizontal reflection, and rotation through 180°, which may further drive historically distinct symbolic systems toward comparable symmetry distributions.

The closeness of the entropy values should nevertheless be regarded as an intriguing empirical convergence rather than evidence for a universal constant. The result may depend on the selected fonts, the categorical treatment of exact symmetry, the limited size of numeral systems, and the adopted graphical conventions. Analysis of additional alphabets, syllabaries, numeral systems, historical scripts, and typefaces will be required to determine whether the observed interval represents a general informational constraint on culturally evolved graphical symbol systems.

The Shannon analysis also reveals that the investigated symbolic and physical systems differ not only in the direction of their symmetry–frequency relationships but also in the diversity of their symmetry-class distributions. The alphabetic and numeral systems generally exhibit comparatively high normalized Shannon entropies, indicating that their symbols are distributed across several symmetry classes. Chinese numerals display the maximally even distribution among the three considered classes. In contrast, the seven-bond colloidal clusters exhibit a strongly concentrated distribution dominated by asymmetric configurations and consequently possess the lowest normalized entropy among the three-class systems. The eight-bond clusters display a broader distribution, although asymmetry remains predominant. Japanese Kanji constitute an especially interesting case: vertical mirror symmetry is globally rare, yielding a relatively low binary entropy, but symmetric characters are disproportionately represented among the most frequent Kanji. Hence, the diversity of symmetry classes and the correlation between symmetry and frequency represent distinct and complementary characteristics of a symbolic or physical system.

## 4. Discussion

### 4.1. Frequency vs. Symmetry

The distributions of alphabetic and numerical symbols are markedly non-uniform. Letter frequencies are primarily determined by the phonological, morphological, and lexical organization of a language, whereas the frequencies of leading numerical figures in many empirical datasets approximately follow Benford’s law [19,21,49,50]. Although the origins and ranges of applicability of Zipf-type and Benford distributions remain subjects of discussion, the unequal occurrence of letters and numerical figures is firmly established [51,52,53].

The principal hypothesis examined in the present study is that symbol frequency is related to the symmetry of its graphical representation. This hypothesis connects two different properties of a symbol: symmetry characterizes the graphical type, whereas frequency characterizes the abundance of its tokens. A symmetry–frequency relationship may therefore be interpreted as a statistical coupling between the type and token aspects of symbolic systems.

The analysis of the mean frequencies of the symmetry classes revealed a positive relationship between letter symmetry and frequency in both English and Russian. The Spearman and Kendall analyses, in which every individual letter was treated as a separate observation, confirmed the positive direction of this relationship. However, the individual-letter correlations did not reach the conventional level of statistical significance. The alphabetic results should therefore be interpreted as indicating a consistent positive tendency rather than providing conclusive statistical confirmation of the proposed relationship.

The regression and rank correlation analyses address different levels of the data. The regression characterizes the mean frequencies of the four symmetry classes, whereas the Spearman and Kendall tests retain the considerable variation among individual letters belonging to the same class. The rank correlation tests consequently provide a more conservative evaluation of the relationship. Taken together, the results suggest that more symmetric letters tend to be more frequent, but they also demonstrate that symmetry alone cannot explain the substantial differences among individual letter frequencies.

Both Spearman’s and Kendall’s tests demonstrated a statistically significant positive association between average graphical symmetry and Benford frequency. The agreement between the linear regression and the two rank correlation analyses indicates that the numerical result does not arise solely from the assumption of a linear relationship.

Nevertheless, the numerical result must be interpreted cautiously. Benford’s law describes the distribution of numerical values in suitable datasets. At the same time, it does not directly describe the frequency with which the corresponding graphical signs were written during the development of numeral systems. Moreover, the average symmetry parameter combines four historically and graphically different numeral systems, and only nine numerical figures are included. The detected association is statistically significant within the investigated dataset, but its historical and cultural origin remains to be established. Future studies should analyze each numeral system separately and compare graphical symmetry with digit frequencies obtained from several independent empirical corpora.

One possible explanation of the observed positive tendency is that frequently used graphemes are subjected to stronger pressure toward economical production and reliable recognition. Even a small reduction in the effort required to write, read, or memorize a highly frequent symbol may produce a considerable cumulative advantage. During the cultural evolution of a writing system, frequently reproduced symbols may therefore be more strongly optimized than rarely used ones.

Symmetry may contribute to this optimization by introducing regularity and redundancy into the graphical structure. When one part of a symmetric glyph is known, another part may be reconstructed through the corresponding geometrical transformation. Symmetry may thus reduce the amount of independent graphical information required to describe or memorize a symbol. In this sense, symmetry provides parsimony in the graphical information necessary for constructing a system of graphemes [17].

Symmetry, however, should not be identified directly with graphical simplicity. A highly symmetric glyph may contain several strokes or possess a long contour, whereas an asymmetric glyph may be constructed from a single simple stroke. Symmetry, visual complexity, writing effort, and ease of recognition are related but distinct characteristics. The observed relationship may therefore be partly mediated by other graphical properties, including stroke number, contour length, curvature, the number of junctions and endpoints, the presence of serifs, and visual similarity to other glyphs [54].

### 4.2. Future Directions

A multivariable analysis incorporating these characteristics would establish whether symmetry remains associated with frequency after graphical complexity has been taken into account. Such an analysis is necessary before symmetry can be regarded as an independent factor in the organization or evolution of writing systems. The direction of causation also remains unresolved. Frequent use may promote the simplification and symmetrization of graphemes. Conversely, symmetric forms may be more easily recognized or reproduced and may therefore be preferentially preserved. Symmetry and frequency may also be indirectly related through historical inheritance or other characteristics of the language and script. The present synchronic study reveals an association, or a tendency toward an association, but cannot establish its causal direction.

A diachronic analysis would provide a more direct test of the evolutionary hypothesis. The symmetry and complexity of the same grapheme could be traced through successive historical stages of an alphabet. If frequently used graphemes systematically acquired symmetry or lost graphical complexity more rapidly than infrequent graphemes, this would constitute stronger evidence that usage frequency influences graphical evolution.

Symmetry may also affect the perception and legibility of symbols [55]. Human vision is particularly sensitive to vertical and horizontal orientations, and vertical mirror symmetry is generally more salient than horizontal symmetry [9]. It is therefore plausible that symmetry contributes to the perceptual stability and rapid recognition of glyphs.

The present study, however, does not directly demonstrate that symmetric letters are more legible. Legibility depends not only on the internal regularity of an individual glyph but also on its distinctiveness from other glyphs. Excessive similarity can hinder recognition. An efficient writing system must balance graphical economy and regularity against the requirement that its symbols remain readily distinguishable.

The proposed connection between symmetry and legibility should therefore be tested experimentally. Recognition accuracy and response time could be measured for glyphs presented under brief exposure, reduced contrast, visual noise, rotation, or peripheral viewing. Symmetry and graphical complexity should be varied independently to determine whether symmetry itself facilitates recognition.

Computer fonts provide internally consistent graphical representations and eliminate variability caused by individual handwriting. At the same time, the symmetry classification of a letter may depend on the selected typeface. Serifs, stroke contrast, curvature, and optical corrections may break an otherwise approximate symmetry. Since different fonts were used for the Latin and Cyrillic alphabets, differences between their results cannot be attributed exclusively to differences between the languages.

Future studies should therefore examine multiple typefaces, including serif, sans-serif, monospaced, and historical fonts. Comparisons between Latin and Cyrillic letters should preferably employ multilingual font families designed according to the same graphical principles. Approximate symmetry could also be measured computationally by comparing each glyph with its reflected and rotated images. A continuous symmetry measure would be more reproducible and informative than a strictly categorical classification. The aesthetic aspect of the fonts should be addressed [56].

### 4.3. Natural vs. Artificial Systems

The comparison with two-dimensional colloidal clusters reveals the opposite tendency: more symmetric configurations are less abundant. This contrast is scientifically interesting, but the two kinds of systems are governed by fundamentally different mechanisms. Grapheme distributions are shaped by language, perception, cultural inheritance, and repeated human use. Colloidal populations are determined by energy, configurational degeneracy, entropy, kinetic accessibility, and experimental preparation. The equilibrium probability of a colloidal configuration may be expressed schematically as

(9) $$P_{i}=\frac{g_{i}exp(-\beta E_{i})}{Z},$$

where $E_{i}$ is the energy of the configuration, $g_{i\;}$ is its configurational statistical weight, $\beta =(k_{B}T{)}^{-1}$, and Z is the partition function. When the configuration energies are approximately equal, relative populations are governed mainly by their statistical weights. Asymmetric structures may be more abundant because more distinguishable arrangements correspond to asymmetric configurations than to highly symmetric ones. Equivalently, asymmetric configurations may occupy a greater volume of configurational space and possess greater configurational entropy. At equal enthalpy, the greater configurational entropy of the less symmetric structures lowers their free energy G=H−TS. Less symmetric configurations may therefore be thermodynamically more favourable. Energy differences and kinetic barriers may also contribute to the measured populations and should not be neglected.

The comparison between graphemes and colloidal clusters should consequently be understood as a contrast between two different mechanisms. In culturally evolved symbolic systems, frequent use may favor graphical economy, regularity, and reliable recognition. In physical cluster systems, increased symmetry may reduce configurational degeneracy and the accessible volume of configuration space. These different mechanisms can naturally generate symmetry–frequency correlations with opposite signs.

The comparison with molecular ligands should likewise be treated as an analogy rather than direct evidence for the grapheme mechanism. The association between molecular order, conformational mobility, and receptor affinity illustrates the importance of configurational freedom in natural systems, but it does not directly explain the historical development of alphabetic symbols.

### 4.4. Type vs. Token

The observed relationships are also relevant to the type–token distinction. Symmetry belongs to the symbol as a graphical type, whereas frequency is measured from the occurrence of its tokens. The results suggest that these two levels may not be statistically independent: repeated production and recognition of tokens may influence the historical form of the type, while the graphical properties of the type may affect the efficiency with which its tokens are processed.

The interpretation in terms of iconicity requires some caution. Iconicity conventionally refers to a motivated resemblance between a sign and its meaning or referent. Alphabetic symbols generally do not visually resemble the sounds they represent. The observed relationship is therefore more directly connected with graphical economy, perceptual optimization, and usage-based evolution. In a broader sense, however, the form of a frequently used symbol may reflect the conditions of its use. The symmetry–frequency relationship could consequently represent a form of use-related motivation of graphical structure.

The present investigation is limited by the small number of alphabets and fonts, the discrete nature of the symmetry score, the unequal sizes of the symmetry classes, the absence of independent measures of graphical complexity, and the synchronic character of the analysis. The numerical study is additionally limited by its small sample and by averaging symmetry across several distinct numeral systems.

Further investigations should include additional alphabetic, syllabic, and logographic scripts; multiple historical and modern typefaces; continuous computational measures of approximate symmetry; and independent measures of graphical complexity and visual distinctiveness. Diachronic studies and controlled perceptual experiments would be especially valuable for determining the causal mechanism behind the observed tendency.

### 4.5. Possible Applications

Other systems of culturally evolved graphical notation—including mathematical symbols, musical notation, programming languages, and chemical symbols—may provide further tests of the proposed relationship. The possible application of symmetry information to the decipherment of ancient scripts is also of interest, particularly when the available corpus is too small for reliable frequency analysis, e.g., the Indus Valley [57] or Rongorongo scripts [58]. Such applications would first require extensive validation across independently developed writing systems.

Overall, the regression and rank correlation analyses consistently reveal a positive symmetry–frequency tendency in the investigated human-made symbolic systems. The individual-letter evidence remains suggestive rather than conclusive, whereas the association found for the numerical figures is statistically stronger. The opposite tendency observed for colloidal clusters may be explained by configurational degeneracy and entropy. Together, these results identify the relationship between graphical symmetry and statistical abundance as a promising interdisciplinary problem connecting cognition, linguistics, information economy, cultural evolution, and statistical physics.

The Shannon analysis reveals that the English and Russian alphabets possess remarkably similar symmetry-class entropies, indicating comparable levels of diversity and evenness in their distributions among the four symmetry classes. This convergence may reflect a common compromise between graphical regularity, which facilitates reproduction and recognition, and graphical distinctiveness, which prevents confusion between letters. Although the result is intriguing, its generality should be tested using additional alphabets and comparable typefaces.

## 5. Conclusions

We investigated the relationship between the graphical symmetry of symbols and their frequency of occurrence in alphabetic, numerical, and logographic systems and compared the observed tendencies with those found in two-dimensional colloidal clusters. The results indicate that symmetry and frequency are not statistically independent in several of the investigated systems. However, the direction, strength, and probable origin of this relationship differ considerably between culturally evolved symbols and physical structures.

The English and Russian alphabets demonstrated a consistent positive tendency: letters possessing a greater number of graphical symmetry operations generally tend to occur more frequently. Spearman’s and Kendall’s rank-correlation coefficients were positive for both alphabets, although the associations did not reach the conventional level of statistical significance. These results should therefore be regarded as suggestive evidence of a symmetry–frequency relationship rather than as its conclusive statistical confirmation.

A stronger result was obtained for the numerical figures. Both Spearman’s and Kendall’s tests demonstrated a statistically significant positive association between the average graphical symmetry of the figures and their leading-digit frequencies predicted by Benford’s law. Within the investigated dataset, more symmetric numerical figures consequently correspond to more frequent leading digits. Because this analysis includes only nine figures and combines symmetry information from four different numeral systems, its historical and cultural interpretation requires further investigation.

The Japanese Kanji provided the strongest statistical evidence for a relationship between graphical symmetry and frequency. The number of vertically mirror-symmetric characters decreases systematically from the most frequent groups toward the less frequent groups. Both Spearman’s and Kendall’s tests confirmed a strong and statistically significant association. Vertically mirror-symmetric Kanji are therefore disproportionately concentrated among the most frequently used characters. This conclusion applies to the frequency-group aggregates used in the present study; analysis of the individual frequencies of all 1000 Kanji would provide a more detailed test.

The Shannon analysis supplied a complementary description of the organization of symmetry in the investigated systems. Whereas the rank-correlation tests determine whether more symmetric symbols tend to be more frequent, the Shannon entropy characterizes the diversity and evenness of the distribution among the available symmetry classes. These two measures address different properties of a symbolic system and should not be interpreted interchangeably.

An intriguing result of the Shannon analysis is the comparatively narrow range of symmetry-class entropies obtained for the English and Russian alphabets and for the Chinese, Western Hindu–Arabic, Roman, and Eastern Arabic numeral systems. Despite their different cultural origins, historical development, and graphical organization, these systems exhibit broadly comparable levels of symmetry-class diversity. The Chinese numerals constitute a limiting case in which the symbols are distributed equally among the three considered symmetry classes.

Part of this convergence arises from the small number of symmetry classes available in the adopted classification. Nevertheless, the similarity remains visible after normalization for the number of classes. It may therefore reflect a common functional compromise operating in culturally evolved graphical systems. Symmetry can promote graphical economy, reproducibility, perceptual stability, memorability, and rapid recognition. Conversely, an excessive concentration of symbols in highly symmetric classes could reduce their graphical distinctiveness and increase the possibility of confusion. Symbolic systems may consequently favor repertoires containing both symmetric and asymmetric forms, thereby maintaining a balance between regularity and distinguishability.

The restricted geometry of planar glyphs may provide an additional explanation. The exact transformations readily realized in written symbols are primarily vertical reflection, horizontal reflection, and rotation through 180°. The limited number of available symmetry operations constrains the possible symmetry-class distributions and may cause historically distinct symbolic systems to converge toward comparable informational characteristics.

The Kanji system presents an especially informative case. Vertical mirror symmetry is globally uncommon among the 1000 investigated characters, and the resulting symmetry-class entropy is considerably lower than those of the alphabets and numeral systems. Nevertheless, the relatively small subset of vertically symmetric Kanji is strongly concentrated among the most frequently used characters. There is no contradiction between these findings. The Shannon entropy shows that the complete Kanji set is dominated by asymmetric characters, whereas the rank-correlation analysis shows that symmetry becomes progressively more prevalent toward the high-frequency end of the corpus. Thus, a system may exhibit low global symmetry diversity while simultaneously possessing a strong symmetry–frequency relationship.

The comparison with colloidal clusters revealed a fundamentally different tendency. In both the seven-bond and eight-bond ensembles, asymmetric configurations are more probable than symmetric configurations. The seven-bond clusters possess a particularly concentrated distribution dominated by the asymmetric class, whereas the eight-bond clusters occupy the available symmetry classes more broadly. Nevertheless, asymmetry remains predominant in both ensembles.

The opposite tendencies observed in symbolic and physical systems probably originate from different underlying mechanisms. Culturally evolved symbols are influenced by repeated production, recognition, learning, graphical economy, perceptual constraints, and historical inheritance. In physical clusters, the populations of different configurations are governed by energy, configurational degeneracy, entropy, kinetic accessibility, and experimental preparation. Asymmetric configurations may be more numerous because they can generally be realized in a larger number of distinguishable ways and may occupy a greater volume of configurational space than highly symmetric configurations.

The combined use of rank correlations and Shannon entropy therefore provides a more complete statistical characterization of symmetry. The correlation coefficients describe whether symmetry is preferentially associated with frequent symbols or configurations, whereas the Shannon entropy describes how broadly the system occupies the available symmetry classes. A strong correlation does not necessarily imply a high symmetry-class entropy, and a high entropy does not necessarily imply a relationship between symmetry and frequency.

The present results suggest that culturally evolved graphical systems may be subject to common geometrical, perceptual, and functional constraints. However, the observed convergence of their symmetry-class entropies should be regarded as an intriguing empirical regularity rather than as evidence for a universal informational constant. Establishing its generality will require the investigation of additional alphabets, syllabaries, logographic scripts, numeral systems, mathematical and musical notation, historical forms, and multiple typefaces. Future studies should also employ individual-symbol frequency data, continuous measures of approximate symmetry, independent measures of graphical complexity, and diachronic analyses. Such investigations may clarify whether symmetry is an independent factor in the cultural evolution of graphical symbols and determine the causal origin of the observed symmetry–frequency relationships.

## Figures and Tables

**Figure 1:**
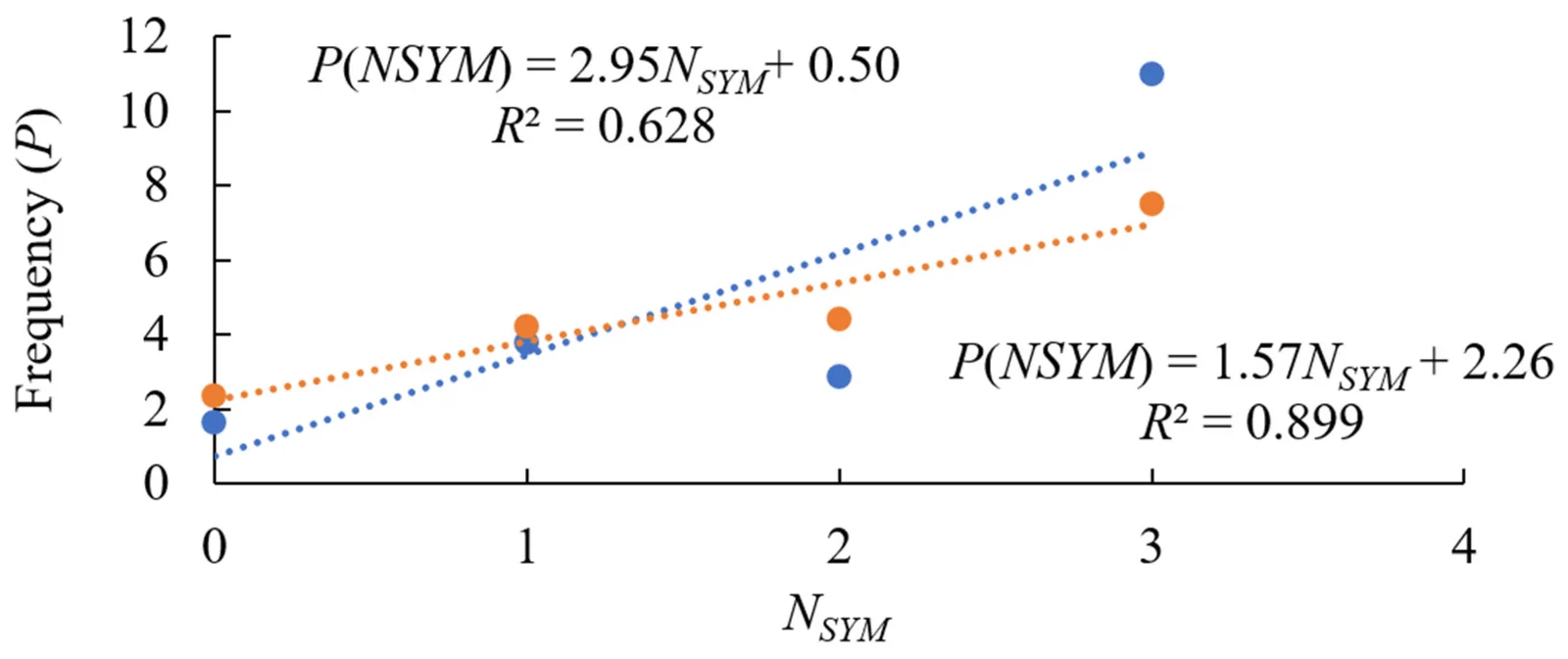
Frequency (%) vs. symmetry for English (orange) and Russian (blue) letters.

**Figure 2:**
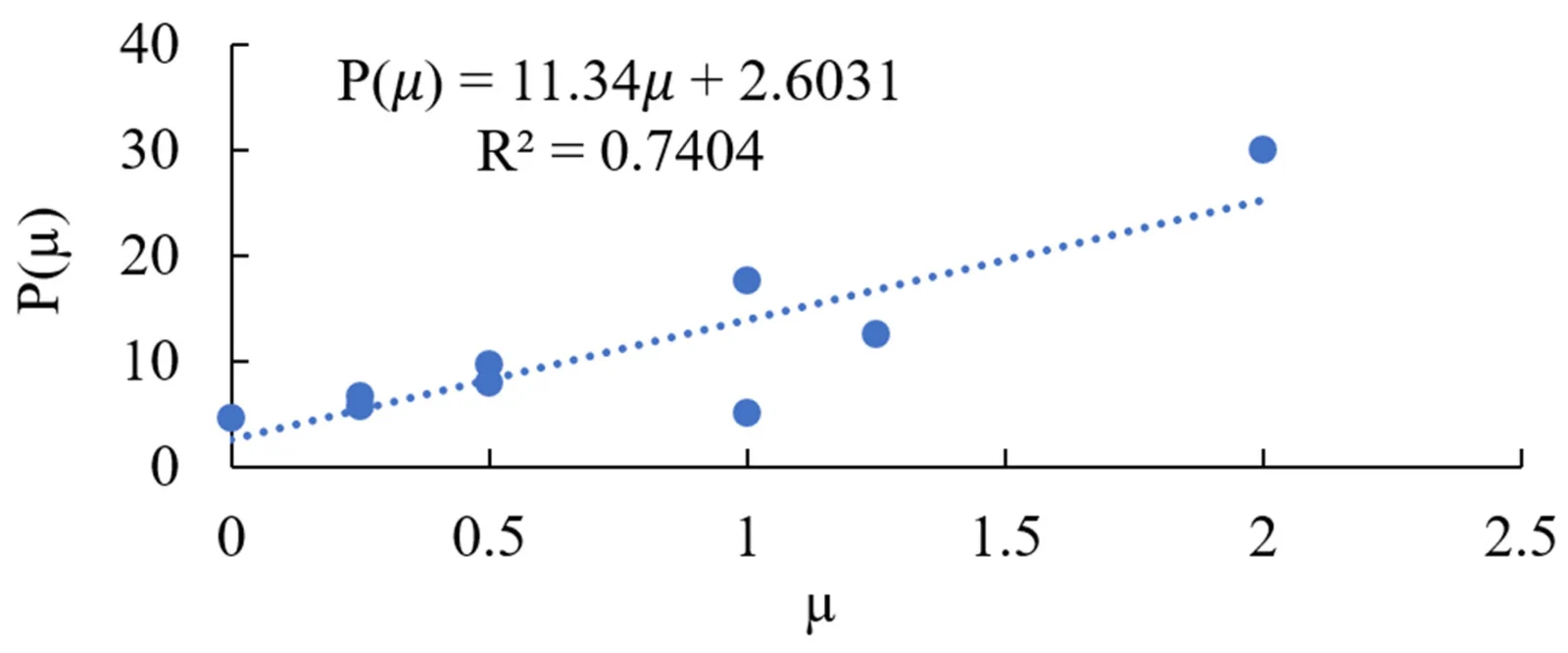
First digit frequency (%) vs. average symmetry parameter *µ* for numerals.

**Figure 3:**
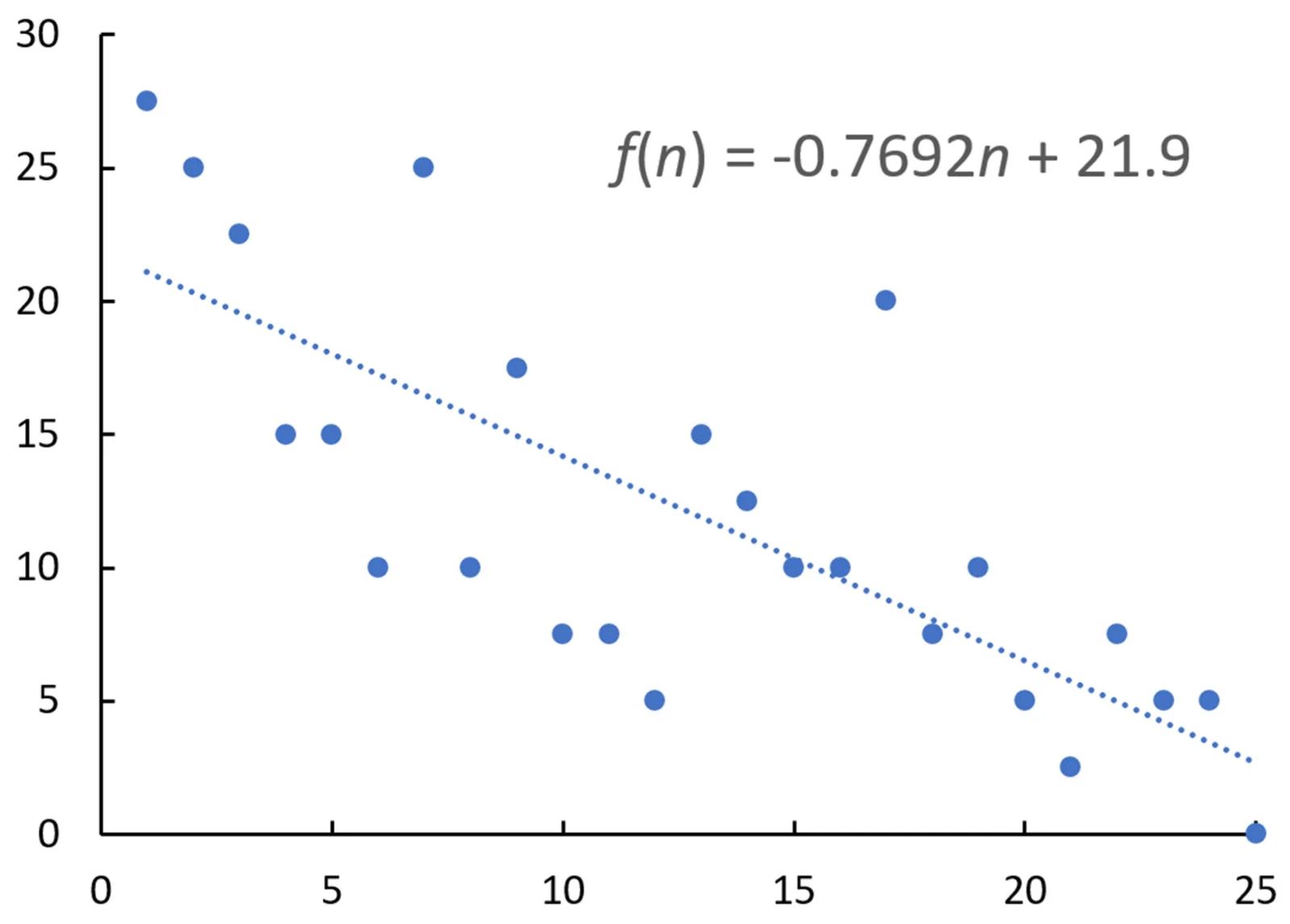
Number of symmetries as a function of frequency rank for Kanji characters.

**Figure 4:**
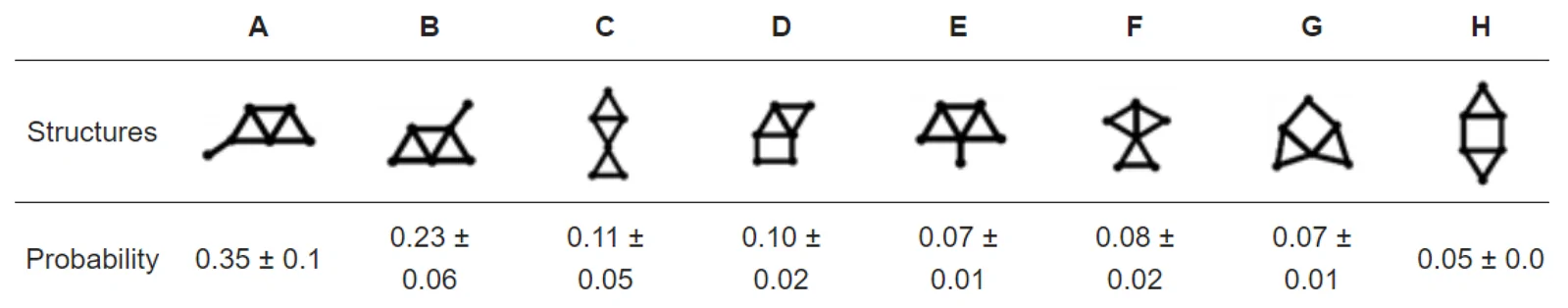
Structures of eight-bond colloidal clusters and their magnitudes of probability distribution [38,39].

**Figure 5:**
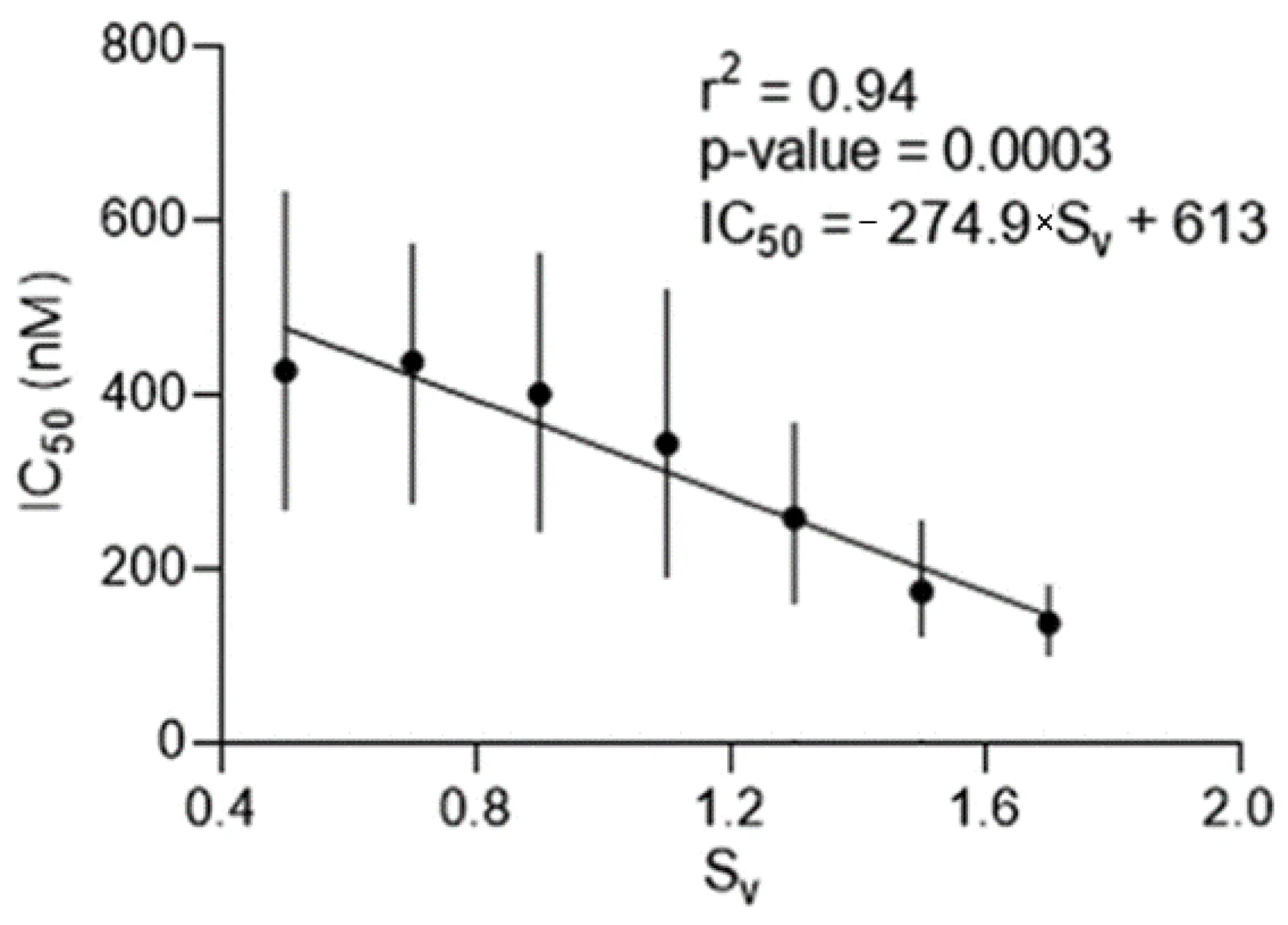
Correlation between the VE and affinity to the SARS-CoV-2 main protease (Mpro) represented by half-maximal inhibitory concentration (IC50), reproduced from [42] with permission.

**Table 1:** Number of symmetry groups in letters of English and Russian (Cyrillic) alphabets.

	Latin Letters	Cyrillic Letters
0	F, G, J, L, P, Q, R,	Б, Г, Ё, Й, Р, Ц, Ч, Щ, Ъ, Ы, Ь, Я
1	A, B, C, D, E, K, M, N, S, T, U, V, W, Y, Z	А, В, Д, Е, З, И, К, Л, М, П, С, Т, Y, Ш, Э, Ю
2	H, I, X	Ж, Н, Х, Ф
3	O	О

**Table 2:** Frequency of letters in English language texts.

Letter	E	T	A	O	I	N	S	H	R	D	L	C	U
Frequency, %	12.7	9.1	8.2	7.5	7.0	6.7	6.3	6.1	6.0	4.3	4.0	2.8	2.8
Letter	M	W	F	G	Y	P	B	V	K	J	X	Q	Z
Frequency, %	2.4	2.4	2.2	2.0	2.0	1.9	1.5	1.0	0.8	0.2	0.2	0.1	0.1

**Table 3:** Frequency of letters in Russian language texts.

Letter	О	Е	А	И	Н	Т	С	Р	В	Л	К	М	Д	П	У	Я
Frequency, %	11.97	8.45	8.01	7.35	6.70	6.26	5.47	4.73	4.54	4.40	3.49	3.21	2.98	2.81	2.62	2.01
Letter	Ы	Ь	Г	З	Б	Ч	Й	Х	Ж	Ш	Ю	Ц	Щ	Э	Ф	Ъ
Frequency, %	1.9	1.74	1.7	1.65	1.59	1.44	1.21	0.97	0.94	0.73	0.64	0.48	0.36	0.32	0.26	0.04

**Table 4:** Number of symmetry operations of four types of numerals from 1 to 9.

Number	Symmetry	Number	Symmetry	Number	Symmetry	Number	Symmetry	Average	*P*
一	2	I	2	I	2	١	2	2	30.1
二	2	2	0	II	2	٢	0	1	17.6
三	2	3	1	III	2	٣	0	1.25	12.5
四	1	4	0	IV	0	٤	1	0.5	9.7
五	0	5	0	V	1	٥	1	0.5	7.9
六	1	6	0	VI	0	٦	0	0.25	6.7
七	0	7	0	VII	0	٧	1	0.25	5.8
八	1	8	2	VIII	0	٨	1	1	5.1
九	0	9	0	IX	0	٩	0	0	4.6

**Table 5:** Number of symmetries in 1000 Japanese Kanji characters arranged by their frequency and listed in groups of 40.

Group	Characters	Symmetries	%
1	日人一大年本中出時行事分会上生国者合自間方見手前場月子地学後入目部長発同新高社的	11	27.5
2	作内動下用代言立定理明体業度通気関対家力表当金実全思物最外話現書名小意性市成来連	10	25
3	今文回開法以戦所化女記主問三道不世取要多知機二野数第持教山心相画使集経正選報民考	9	22.5
4	先期近情員利加面点水無在変次公初決安原品結解政東活語題保特信向車別私受平界海重引	6	15
5	議付続真能元強田都組感電調指制少身和治何校男産口有説十楽示切約円県直確番川送交際	6	15
6	空進得神売件務勝権食設運認必参位過町式置料流広北天可論共五支果氏終味計線聞死店始	4	10
7	村万反島常木様半投状容放院予格着土住美屋台四応区判形転団基朝総白音役係工葉由西足	10	25
8	他改伝軍止風起質仕配育張告資術声好親構頭府落優供士京済八義求検然石打価門再良乗局	4	10
9	任古種光観注営映両限想帰読夫色号残態案達職追字存写演断査米愛南急消命提側統商球科	7	17.5
10	建備首族条登研象呼早千九太図病路造悪馬技協個害念待収例増去各護官等派究規夜験館歩	3	7.5
11	非細母像型割器返難試室証歌史録戸客助単視勢医素比火移党識準師花失段王域武量争満除	3	7.5
12	福六井差製宮降類州殺若歴百編望守買独周園値負英評処系右七銀深横談防走接速管兵座策	2	5
13	根境父異黒友復程率申衛青末警赤展働答領笑顔左挙松鉄算紙毎減察修導低辺駅退覚費春景	6	15
14	帯疑旅極宅完版未曲担階週省専賞装著材寄飛姿補効労谷隊習農橋株具居委源船久述整将城	5	12.5
15	財夏席願児精競故健佐興織絶波適積熱紀級革秋敗薬破標休苦囲温節岡洋税森背芸便遠険庫	4	10
16	幸宿蔵巻探額星裁許司富授軽激推並従午遺香血順課林給板略訪雑陸角寺港留丸印玉君志短	4	10
17	属静崎模豊遊服因清余河圧酒拡康障息列危密盛皇街照冷講飲央肉章逆刻喜責老泉草散我布	8	20
18	旧絵輪裏庭衆昨植焼養訳博劇妻曜候針夢婦罪亡阪諸築練善創鳥仲堂幹茶似就雨徒航討承典	3	7.5
19	岩誌禁採乱否令測油傷厳固犯岸忘輸幕陽納欲宇複筆辞徳困則永痛秘池筋宗札貴延益里簡停	4	10
20	枚倍己脳羽昭勤敵票染暴片刊混易季底功坂群祭希折射夕閉快暮層厚億樹竹欠暗弱閣庁奈倉	2	5
21	卒雪弟毛批宣賀兄栄救績純副骨仮聖晴券魚覧礼届操憲仏律署束沖迷宝飯損丁犬盟災吸冬鹿	1	2.5
22	頂貨郷至均悲借謝縮郵歯腹荷包臣湯洗衣翌燃牛臨昼窓舎黄幼童砂誤鳴郡眼奏菜昔誕酸胸乳	3	7.5
23	貸沿宙雲縄皮塩秒耳詩努祖賛液招勉箱梅揮句姉矢敬捨浅慣看漢浴熊忠緑桜尊虫豆祝妹旗泣	2	5
24	賃訓紅寒勇干湖才銭穴毒潮柱詞鏡誠仁唱帳孫漁俳臓貧枝畑炭拝預晩岐牧序灯飼刀暖冊械孝	2	5
25	鼻粉往棒氷卵熟刷墓糸貯縦泳潟脈肥貿麦弁兆糖梨垂朗恩埼暑蒸奮巣拾銅鉱径綿舌寸磁灰茨	0	0

**Table 6:** Average symmetry parameter (based on the four types of numerals) and the first digit frequency.

Numeral	1	2	3	4	5	6	7	8	9
Average symmetry, µ	2.0	1.0	1.25	0.5	0.5	0.25	0.25	1	0
Frequency (Benford’s Law), %	30.1	17.6	12.5	9.7	7.9	6.7	5.8	5.1	4.6

**Table 7:** Frequency vs. Symmetry for 7-bond and 8-bond colloidal clusters (based on [38,39]).

Seven-Bond Colloidal Cluster		Eight-Bond Colloidal Cluster	
Symmetry	Probability, %	Symmetry Operations, *N_SYM_*	Probability, %
0	88.4	0	65
1	9.6	1	31
2	2	2	4
$P\left(N_{SYM}\right)=-43.2N_{SYM}\;+\;$76.53	$R^{2}=0.815$	$P\left(N_{SYM}\right)=-30.5N_{SYM}+63.83$	$R^{2}=0.996$

**Table 8:** Spearman and Kendall analysis for the data related to English and Russian alphabets and numerical figures.

Symbolic System	Number of Symbols, *n*	Spearman’s $\rho_{s}$	Spearman’s *p*	Kendall, $\tau_{b}$	Kendall *p*-Value
English alphabet	26	0.337	0.092	0.268	0.094
Russian alphabet	32	0.281	0.120	0.245	0.083
Numerical figures, 1–9	9	0.760	0.0176	0.667	0.0148
Japanese Kanji, 40-character frequency groups	25	0.770	$6.72\times 10^{-6}$	0.635	${1.74\times 10}^{-5}$

**Table 9:** Shannon entropy of the symmetry-class distributions in the investigated systems.

System	Symmetry-Class Distribution	$H_{class}$, Nats	Normalized Entropy, $H_{norm}$
English alphabet	(7, 15, 3, 1)	1.0451	0.7539
Russian alphabet, including Ё	(12, 16, 4, 1)	1.0806	0.7795
Chinese numerals	(3, 3, 3)	1.0986	1.0000
Western Hindu–Arabic numerals	(6, 1, 2)	0.8487	0.7725
Roman numerals	(5, 1, 3)	0.9369	0.8528
Eastern Arabic numerals	(4, 4, 1)	0.9650	0.8783
Japanese Kanji: symmetric/asymmetric	(119, 881)	0.3649	0.5265
Seven-bond colloidal clusters	(0.884, 0.096, 0.020)	0.4122	0.3752
Eight-bond colloidal clusters	(0.65, 0.31, 0.04)	0.7718	0.7026

## Data Availability

The data presented in this study are available on request from the corresponding author.

## Appendix A

This Appendix briefly explains the non-parametric statistical methods used to evaluate the relationship between symbol symmetry and frequency. The explanation is intended for readers who may not be familiar with rank correlation analysis.

The symmetry score of an alphabetic symbol takes one of only four values: $N_{SYM}=0,1,2,3.$ These values indicate the number of considered graphical symmetries: vertical reflection, horizontal reflection, and rotation by $180^{\circ }$. Consequently, many letters have the same symmetry score. The letter frequency distributions are strongly non-Gaussian: a few letters occur very frequently, whereas many are relatively rare. Under these conditions, the ordinary linear correlation (see Figure 1) is not the best method for evaluating individual symbols. As opposed to that, Spearman’s and Kendall’s methods are non-parametric rank correlation tests. They do not require normally distributed variables or a linear relationship. They test whether higher values of one variable generally correspond to higher or lower values of the other variable. Consequently, they can be used to test whether symbols with greater graphical symmetry are more likely to be more frequent. Each letter or numerical figure is treated as a separate observation.

Spearman’s rank correlation coefficient measures the strength and direction of a monotonic relationship between two variables [47]. The calculation begins by replacing the original values with their ranks. For example, the letters are ranked according to (i) their symmetry scores (ii) their frequencies. The average rank was assigned when several observations had the same value (many letters had identical symmetry scores). Spearman’s coefficient is the ordinary Pearson correlation coefficient calculated between the two sets of ranks:

(A1) $$\rho_{s}=\frac{cov(R_{S},R_{P})}{\sigma_{R_{S}}\sigma_{R_{P}}},$$

where $R_{S}$ is the rank of the symmetry score and $R_{P}$ is the rank of frequency. When there are no tied ranks, the coefficient can also be written as

(A2) $$\rho_{s}=1-\frac{6\sum_{i=1}^{n}d_{i}^{2}}{n\left(n^{2}-1\right)},$$

where $d_{i}\;$ is the difference between the two ranks assigned to observation i, and n  is the number of observations. Because the present data contain numerous ties, the correlation-between-ranks definition was used. The coefficient lies in the interval $-1\leq \rho_{s}\leq 1.$ Positive values indicate that more symmetric symbols tend to be more frequent, while negative correlation indicates the opposite. Provided that frequency generally increases with symmetry, Spearman’s coefficient will detect a positive monotonic relationship.

Kendall’s method, denoted by τ, evaluates the ordering of all possible pairs of observations. Consider two letters, *i* and *j*, with symmetry scores $S_{i}$ and $S_{j}\;$ and frequencies $P_{i}$ and $P_{j}$. The pair is concordant if the more symmetric letter is also the more frequent letter $(S_{i}-S_{j})(P_{i}-P_{j})>0$ and discordant otherwise.

For tied pairs, a correction was used

(A3) $$\tau_{b}=\frac{C-D}{\sqrt{\left(C+D+T_{S})(C+D+T_{P}\right)}},$$

where C is the number of concordant pairs, D  is the number of discordant pairs, $T_{S}$ and $T_{P}$ are the number of pairs tied only in symmetry and in frequency. Like Spearman’s coefficient, Kendall’s coefficient is in the range $-1\leq \tau_{b}\leq 1.$ A positive value means that concordant pairs predominate, whereas a negative value means that discordant pairs predominate.

In the absence of ties, Kendall’s coefficient represents the difference between the probability of selecting a concordant pair and the probability of selecting a discordant pair:

(A4) τ=P(concordant pair)−P(discordant pair).

Spearman’s and Kendall’s coefficients evaluate the same general hypothesis but use different mathematical approaches. Spearman’s coefficient compares the ranks assigned to individual observations. Kendall’s coefficient directly compares all possible pairs of observations. Kendall’s $\tau_{b}$ is particularly well suited to data containing numerous ties, whereas Spearman’s coefficient is commonly used and readily interpretable as a rank correlation. Agreement between the two tests indicates that the conclusion is not an artifact of one particular statistical procedure. In the present study, the signs of the Spearman and Kendall coefficients agree for every investigated dataset. The numerical values of $\rho_{s}$ and $\tau_{b}\;$ should not be expected to be identical. Kendall’s coefficients are commonly smaller in absolute magnitude because they are defined from differences between concordance and discordance probabilities rather than correlations between ranks.

For each test, the null hypothesis ($H_{0}$) is that symmetry and frequency are not monotonically associated. The alternative hypothesis ($H_{1}$) is that symmetry and frequency are monotonically associated. A two-sided test allows for either a positive or a negative relationship. It is more conservative than a one-sided test and avoids assuming in advance that the association must occur in the proposed direction. The p-value expresses how unusual the observed rank association would be if the null hypothesis were true. At the conventional significance level α=0.05, the null hypothesis is rejected when p<0.05. A result with p≥0.05 does not mean that no relationship exists. Instead, it means that the available data do not provide sufficiently strong evidence to reject the null hypothesis at the selected significance level. A moderate correlation may fail to reach significance in a small sample, whereas a weak correlation may become significant in a very large sample.

## References

[1] Bornstein M.H., Gross C., Wolf J.Z. (1978). Perceptual similarity of mirror images in infancy. Cognition.

[2] Pizlo X., de Barros J.A. (2021). The Concept of Symmetry and the Theory of Perception. Front. Comp. Neurosci..

[3] Walsh E., Moreira C., Longo M.R. (2024). Opposite size illusions for inverted faces and letters. Cognition.

[4] Green E.G. (2023). Perceptual constancy and perceptual representation. Anal. Philos..

[5] Bormashenko E. (2020). Entropy, Information, and Symmetry: Ordered is Symmetrical. Entropy.

[6] Van Fraassen B.C. (1989). Laws and Symmetry.

[7] Weyl H. (1989). Symmetry.

[8] King M., Meyer G.E., Tangney J., Biederman I. (1976). Shape constancy and a perceptual bias towards symmetry. Percept. Psychophys..

[9] Morin O. (2018). Spontaneous Emergence of Legibility in Writing Systems: The Case of Orientation Anisotropy. Cogn. Sci..

[10] Hutton C. (1990). Abstraction; Instance: The Type-Token Relation in Linguistic Theory.

[11] Lyons J. (1977). Semantics.

[12] Zipf G.K. (1935). The Psychobiology of Language.

[13] Clauset A., Shalizi C.R., Newman M.E.J. (2009). Power-Law Distributions in Empirical Data. SIAM Rev..

[14] Koshevoy O., Milton H., Morin O. (2023). Zipf’s Law of Abbreviation holds for individual characters across a broad range of writing systems. Cognition.

[15] Sheedy J.E., Subbaram M.V., Zimmerman A.B., Hayes J.R. (2005). Text Legibility and the Letter Superiority Effect. Hum. Factors.

[16] Gibson E., Futrell R., Piantadosi S.T., Dautriche I., Mahowald K., Bergen L., Levy R. (2019). How efficiency shapes human language. Trends Cogn. Sci..

[17] Gilevich A., Frenkel M., Shoval S., Bormashenko E. (2024). Time Evolution of the Symmetry of Alphabet Symbols and Its Quantification: Study in the Archeology of Symmetry. Symmetry.

[18] Revesz P.R., Viana V., Nagy D., Xavier J., Neiva A., Ginoulhiac M., Mateus L., Varela P. (2022). The development and role of symmetry in ancient scripts. Symmetry: Art and Science|12th SIS Symmetry Congress.

[19] Fewster R.M. (2009). A simple explanation of Benford’s Law. Am. Stat..

[20] Miller S.J. (2015). Benford’s Law: Theory and Applications.

[21] Whyman G., Shulzinger E., Bormashenko E. (2016). Intuitive considerations clarifying the origin and applicability of the Benford law. Results Phys..

[22] Tanchip C., Yu L., Xu A., Xu Y. (2020). Inferring symmetry in natural language. Findings of the Association for Computational Linguistics: EMNLP 2020.

[23] Gleitman L.R., Senghas A., Flaherty M., Coppola M., Goldin-Meadow S. (2019). The emergence of the formal category “symmetry” in a new sign language. Proc. Natl. Acad. Sci. USA.

[24] Winter Y. (2018). Symmetric predicates and the semantics of reciprocal alternations. Semant. Pragmat..

[25] Tversky A., Gati I. (1978). Studies of similarity. Cognition and Categorization.

[26] Daggumati S., Revesz P.Z. (2023). Convolutional Neural Networks Analysis Reveals Three Possible Sources of Bronze Age Writings between Greece and India. Information.

[27] Kim Y., Allassonnière-Tang M., Miton H., Morin O. (2025). The phonology of letter shapes: Feature economy and informativeness in 43 writing systems. J. Mem. Lang..

[28] Derpsch Y., Tyson-Carr J., Rampone G., Bertamini M., Makin A.D.J. (2024). Event-related potentials reveal a robust response to visual symmetry in unattended visual regions. NeuroImage.

[29] Moreau R., Alp N., Clarke A.D.F., Freud E., Kohler P.J. (2025). Visual search efficiency is modulated by symmetry type and texture regularity. J. Vis..

[30] Tyson-Carr J., Bertamini M., Rampone G., Jones A., Makin A.D.J. (2025). When do we find a third neural response to visual symmetry?. Cortex.

[31] Leland R. (2000). Cryptological Mathematics.

[32] Ляшевская О.Н., Шаров С.А. (2009). Новый Частотный Словарь Русской Лексики 2009.

[33] Kandrac P. (2021). 2242 Kanji Frequency List ver. 1.1. https://en.wikipedia.org/wiki/Ky%C5%8Diku_kanji.

[34] Mekonnen A.M., Joshi R.M. (2026). Visual complexity and frequency of graphemes in Amharic: Implications for dyslexics and dysgraphics. Cogn. Process..

[35] Fedorets A.A., Bormashenko E., Dombrovsky L.A., Nosonovsky M. (2020). Symmetry of small clusters of levitating water droplets. Phys. Chem. Chem. Phys..

[36] Janai E., Schofield A.B., Sloutskin E. (2012). Non-crystalline colloidal clusters in two dimensions: Size distributions and shapes. Soft Matter.

[37] Lim M., Souslov A., Vitelli V., Jaeger H. (2019). Cluster formation by acoustic forces and active fluctuations in levitated granular matter. Nat. Phys..

[38] Nosonovsky M., Roy P. (2020). Scaling in Colloidal and Biological Networks. Entropy.

[39] Perry R.W., Holmes-Cerfon M.C., Brenner M.P., Manoharan V.N. (2015). Two-Dimensional Clusters of Colloidal Spheres: Ground States, Excited States, and Structural Rearrangements. Phys. Rev. Lett..

[40] Crocker J.C. (2010). Turning Away from High Symmetry: Light microscopy studies of cluster formation by colloidal particles show that less-symmetrical structures are favored under equilibrium conditions. Science.

[41] Meng G., Arkus N., Brenner M., Manoharan V.N. (2010). The Free-Energy Landscape of Clusters of Attractive Hard Spheres. Science.

[42] Shityakov S., Aglikov A.S., Skorb E.V., Nosonovsky M. (2023). Voronoi Entropy as a Ligand Molecular Descriptor of Protein–Ligand Interactions. ACS Omega.

[43] Garrod S., Fay N., Lee J., Oberlander J., Macleod T. (2007). Foundations of representation: Where might graphical symbol systems come from?. Cogn. Sci..

[44] Nöth W. (1999). Peircean Semiotics in the Study of Iconicity in Language. Trans. Charles S. Peirce Soc..

[45] Croft W. (1995). Autonomy and Functionalist Linguistics. Language.

[46] Rao V.C.S. (2021). Cognitive Linguistics: An Approach to the Study of Language and Thought. J. Res. Sch. Prof. Engl. Lang. Teach..

[47] Heckert N.A., Filliben J.J., Croarkin C.M., Hembree B., Guthrie W.F., Tobias P., Prinz J. (2002). NIST/SEMATECH e-Handbook of Statistical Methods.

[48] Conover W.J. (1999). Practical Nonparametric Statistics.

[49] Cerqueti R., Lupi C. (2023). Severe testing of Benford’s law. Test.

[50] Geyer C.L., Williamson P.P. (2004). Detecting fraud in data sets using Benford’s law. Commun. Stat.–Simul. Comput..

[51] Ferrer-i-Cancho R., Elvevag B. (2010). Random Texts Do Not Exhibit the Real Zipf’s Law-Like Rank Distribution. PLoS ONE.

[52] Piantadosi S.T. (2014). Zipf’s word frequency law in natural language: A critical review and future directions. Psychon. Bull. Rev..

[53] Popescu I.I., Altmann G., Köhler R. (2010). Zipf’s law—Another view. Qual. Quant..

[54] Dering B., Wright D., Gheorghiu E. (2014). The interaction between luminance-polarity grouping and symmetry axes on the ERP responses to symmetry. Vis. Neurosci..

[55] Wang M., Yang J., Yu Y., Wu Q., Wu F. (2025). Neural mechanisms of symmetry perception: Hemispheric specialization and the impact of noise on reflection symmetry detection. Front. Neurosci..

[56] Verrelli C.M., Caprioli L., Romagnoli C., El Aryashi M., Ryszczuk M., Losa M. (2026). Golden Ratio and Cybernetic Aesthetics for Hidden Patterns in Repetitive Human Movements: Golden-faux-sinus as a Two-Dimensional Optimization Model. Biol. Cybern..

[57] Kak S.C. (1988). A frequency analysis of the Indus script. Criptologia.

[58] Korovina E. (2026). A Computational Evaluation of Syllabic Hypotheses for Rongorongo: Evidence from N-gram Analysis. Proceedings of the Fourth Workshop on Language Technologies for Historical and Ancient Languages (LT4HALA 2026) @ LREC 2026.

